# Hepatotoxicity of Antimycotics Used for Invasive Fungal Infections: In Vitro Results

**DOI:** 10.1155/2017/9658018

**Published:** 2017-04-04

**Authors:** Sandra Doß, Heike Potschka, Fanny Doß, Steffen Mitzner, Martin Sauer

**Affiliations:** ^1^Fraunhofer Institute for Cell Therapy and Immunology, EXIM, Rostock, Germany; ^2^Department of Anesthesiology and Intensive Care Medicine, University Hospital of Rostock, Schillingallee 35, Rostock, Germany; ^3^Department of Medicine, Division of Nephrology, University Hospital of Rostock, Schillingallee 35, Rostock, Germany

## Abstract

*Purpose.* Drug-induced liver injury (DILI) is the most common cause of liver injury and a serious clinical problem; antimycotics are involved in approximately 3% of all DILI cases. The hepatotoxicity of many drugs, including the antimycotics, is poorly screened in human models.* Methods.* In a standardized assay the cytotoxicity on hepatocytes of different concentrations (*C*max, 5x *C*max, and 10x *C*max) of the antimycotics used for systemic infections was tested. Anidulafungin (ANI), liposomal amphotericerin B (L-AmB), caspofungin (CASPO), fluconazole (FLUCO), and voriconazole (VORI) were incubated with HepG2/C3A cells. After incubation, the viability of cells (XTT test, LDH release, trypan blue staining), the synthesis of albumin, the cytochrome 1A2 activity, and the cell death (DNA fragmentation) were determined. Kruskal-Wallis and Mann–Whitney tests were used for statistical analyses.* Results.* L-AmB, ANI, and CASPO showed a mild hepatotoxicity in the *C*max concentrations. Higher concentrations of anidulafungin led to a severe impairment of hepatocyte viability and function. The azoles FLUCO and VORI had a higher hepatotoxic potential in all concentrations.* Conclusion.* Antimycotics, especially azoles, used for systemic infections should be given with caution in patient with liver insufficiency or liver failure or high risk for this; therefore, therapeutic drug monitoring should be used. Further studies with this approach are encouraged.

## 1. Introduction

Drug-induced liver injury (DILI) is often responsible for hepatic dysfunction, the termination of drug development, or postmarket withdrawal of approved drugs [1–3]. DILI is a rare but serious clinical problem that is associated with significant morbidity and mortality. It is generally categorized as “idiosyncratic” or “intrinsic.” These two forms of DILI are contrasted in their manifestation and diagnosis. Acetaminophen (APAP) is a common example for intrinsic hepatotoxins [4]; an APAP overdosage is the most frequent cause of drug-induced acute liver failure. It is dose-dependent and is reproducible in animal models [2]. Unfortunately, the underlying molecular mechanisms are not completely understood. Idiosyncratic DILI is typically rare and less reproducible. It occurs, when individuals are exposed to environmental or metabolic predispositions [5, 6].

More than 1000 drugs have been associated with DILI; DILI mimics severe liver injury and acute liver failure at therapeutic dose [7]. The early identification of hepatotoxic molecules and the prediction of DILI are main objectives of pharmacogenomics [3], but there is no “universal” in vitro screening approach. The current preclinical experimental models, including in vitro tests or the regulatory animal toxicity studies, might be insufficient for detecting certain types of human hepatotoxins [8]. These experimental models have strengths and weaknesses. After intensive and ongoing research, the prediction of human DILI using in vitro models remains relatively reliable. Animal models, however, are limited in detecting of human DILI. Data generated in animal models may not be translated into humans; moreover, these data may not be sufficient to predict DILI in humans, because there are species-specific differences in metabolism and pharmacokinetics [9, 10]. In a retrospective study of 150 drugs, only 43% of human toxicities had been correctly predicted with animal tests compared to clinical experience [11].

Approximately 3% of DILI cases are caused by antimycotics (including acute liver failure) [12]. Invasive fungal infections increase and are associated with substantial morbidity and mortality rates, especially in immunosuppressed patients. Currently, there are only 14 invasive antimycotics available, which are poorly investigated for drug-induced hepatotoxicity [13, 14]. Significant differences exist between the triazoles in regard to pharmacokinetics and pharmacodynamics, drug-drug interactions, concerning efficacy, and toxicity [15]. There is an increasing prevalence of resistant fungi [16, 17]. For the effective personalized management of invasive fungal infections it is important to understand potential interactions and toxicities associated with antimycotics; moreover, it is important to understand the pharmacokinetics and pharmacodynamics of the antimycotics. Therefore, therapeutic drug monitoring (TDM), in combination with clinical assessment of the therapeutic effect, may help to optimize treatment results [18]. The aim of the presented study was to determine the hepatotoxicity of antimycotics used for systemic infections with human hepatocytes (HepG2/C3A) in an established in vitro cytotoxicity screening model [19–22].

## 2. Material and Methods

### 2.1. Cell Cultures

HepG2/C3A (ATCC, ref. number CRL-10741) were cultured in Dulbecco's modified Eagle's medium (DMEM, GIBCO Life Technologies, Darmstadt, Germany) supplemented with 10% fetal bovine serum (FBS, PAA Laboratories, Pasching, Germany), 1% of 200 mM L-glutamine (PAA), and 1% of antibiotics solution (Penicillin G: 10.000 IE/ml/Streptomycin: 10 mg/ml; PAA). Cultures were maintained at 37°C in a 5% CO_2_ humidified incubator. Cell concentration and vitality were assessed by trypan blue staining technique.

### 2.2. Drug Solutions

The lowest concentration of the different antifungals was the mean plasma level after induction of an i.v. therapy (*C*max) of anidulafungin (1x 100 mg daily, ANI), caspofungin (1x 50 mg daily, CASPO), fluconazole (2x 400 mg, FLUCO), voriconazole (1x 200 mg, VORI), and liposomal amphotericerin B (5 mg/kg/day i.v., L-AmB). Additionally to the *C*max, we tested two higher concentrations of the drugs (5x *C*_max_, 10x *C*_max_): ANI (7.5, 37.5, 75 *µ*g/ml; 2.25, 11.25, 22.5 mmol; Pfizer, NYC, USA) [23], CASPO (1, 5, 10 *µ*g/ml; 0.2, 1.0, 2.0 mmol; MSD, NJ, USA) [24], FLUCO (2.5, 12.5, 25 *µ*g/ml; 4.5, 22.5, 45 mmol; ratiopharm, Ulm, Germany) [25], VORI (9, 45, 90 *µ*g/ml; 0.25, 1.25, 2.5 mmol; Pfizer, NYC, USA) [26], and L-AmB (22.9, 112.5, 229 *µ*g/ml; 5.725, 28.625, 57.25 mmol; Gilead, CA, USA) [27].

### 2.3. Cytotoxicity on Hepatocytes Assay, pH Measurement

The antimycotics were tested in an established microtiter plate assay for screening of hepatotoxicity [19–22]. HepG2/C3A cells were seeded in 24-well plates at 500.000 cells/well and were incubated for 72 hours with the different drugs in 1 ml medium or heparinized plasma from healthy volunteers (pooled plasma). Subsequently, fresh medium with the different concentrations of the antifungal were incubated with the cells for 72 hours again. Negative control served medium or plasma without agents and acetaminophen (APAP, 15,24 mM in medium, Sigma Aldrich, Seelze, Germany) was used as positive control. Each test batch was performed as a triplicate. The pH values (Radiometer, ABL, Willich, Germany) were screened before and after 72 hours and after 144 hours in the cell culture supernatant.

The following tests were carried out after 144 hours using the cells suspension or the cell culture supernatants.

Trypan blue exclusion assay (0.4% (w/v); Sigma, Seelze, Germany) was used to determine the vitality and the number of cells with a C-Chip Neubauer improved haemocytometer (peqlab, Erlangen, Germany).

Albumin was determined nephelometrically from 0.2 ml cell culture medium supernatant (Immage 800, Beckman Coulter GmbH, Germany) [28].

Plasma membrane integrity was determined by measuring lactate dehydrogenase (LDH) after 72 and 144 hours of incubation [29]. 0.2 ml cell culture medium supernatant was photometrically measured by the change in the absorbance at 340 nm with the automated chemistry analyzer (Cobas Mira, Roche, Mannheim, Germany) according to the optimized standard method of the Deutsche Gesellschaft für Klinische Chemie (DGKC).

Cell viability was also determined using the 2,3-bis-(2-methoxy-4-nitro-5-sulfophenyl)-2H-tetrazolium-5-carboxanilide inner salt (XTT, Roche Diagnostics GmbH, Mannheim, Germany), which is a colorimetric assay and is based on the ability of metabolic active cells to reduce the soluble tetrazolium salt (XTT) into an insoluble formazan with mitochondrial dehydrogenases [30, 31]. At the start of the XTT-determination 2 × 100 *µ*l cell suspension as duplicate was transferred to a transparent 96-well plate. After adding 100 *µ*l XTT-reaction reagent per well the increased formazan absorbance was read at a wavelength of 450 nm on a microplate reader (Anthos Reader 2001, Anthos Labtec Instruments, Austria) after one hour.

The activity of cytochrome P450 1A2 was determined by means of O-deethylization of 7- ethoxyresorufine to resorufin (EROD) [32] according to the method of Donato et al. [33]. Before measurement, hepatocytes stimulated with methylcholanthrene (3-MC, Sigma Aldrich, Seelze, Germany) for 72 hours were treated with 8 *μ*M ethoxyresorufin (Molecular Probes, Eugene, USA) and 10 *μ*M dicumarol (Sigma Aldrich, Seelze, Germany) for 1 h (37°C, 5% CO_2_). Resorufin concentration in the supernatants was measured at 530 nm (excitation) and 584 nm (emission) using a fluorescence multiwell plate reader (Fluoroskan Ascent Lab Systems, Vienna, USA). Concentrations were estimated against a resorufin standard curve (0 pmol, 10 pmol, 20 pmol, 40 pmol, and 80 pmol).

Fluorescence-activated cell sorting (FACS) analysis of DNA fragmentation was examined by the terminal deoxynucleotidyltransferase- (TdT-) mediated dUTP nick end labeling (TUNEL) method using the commercial APO-Direct Kit (BD Pharmingen, San Diego, CA, USA). Terminal deoxynucleotidyl transferase adds FITC-dUTP to each 3′-hydroxyl end of fragmented DNA, making it possible to measure the fragmentation of the DNA by the intensity of fluorescence.

The assay was performed following manufacturer's instructions. After treatment, cells were fixed with 1% paraformaldehyde in PBS (4°C, 30 min), washed in PBS, permeabilized with ice cold 70% ethanol, and stored at −20°C overnight. The next day, after staining the cells the intensity of the fluorescence was determined by FACS analysis (FACS Calibur). In total, 5.000 events were collected per test sample. The results were analyzed by CellQuest software (both from BD Biosciences Systems, San Jose, CA, USA). The percentage of TUNEL negative and positive cells was presented.

### 2.4. Statistics

Statistical analysis was done with the Statistical Package for the Social Sciences (SPSS, Chicago, USA). Statistical differences between groups were analyzed using the Kruskal-Wallis test, because data were not normally distributed. Afterwards, the Mann–Whitney *U* test for pairwise comparison was used. The results are expressed as median, 25th and 75th quartiles. A *p* value of <0.05 was considered as significant.

## 3. Results

### 3.1. Effects of Antimycotics on Cell Proliferation and Vitality

**Figure 1:**
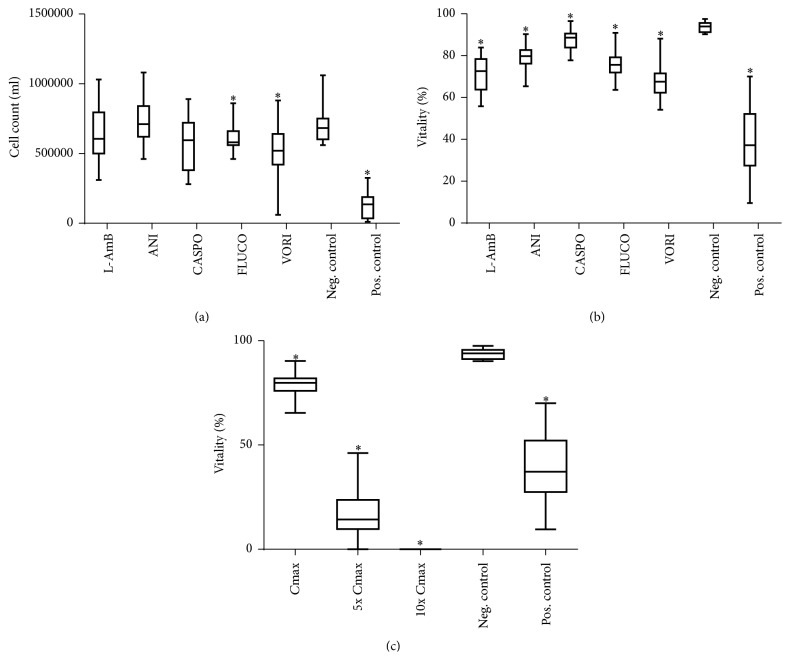
Effects of antimycotics agents on cell vitality and cell count in medium. Cells in culture were treated with 5.7 mM liposomal amphotericerin B, 2.25 mM anidulafungin, 0.2 mM caspofungin, 4.5 mM fluconazole, and 0.25 mM voriconazole and untreated (negative control) and 15.24 mM APAP (positive control) for 144 hours. Cells were counted in each treatment ((a), *C*max) and percent cell vitality was determined with trypan blue staining ((b), *C*max). Cell vitality at different doses of anidulafungin was further measured by trypan blue staining (c). Results are expressed as median and 25th/75th percentile, *n* = 20 (biological and technical repeats). *∗* indicates significance of *p* < 0.05 against the negative control.

The cell count was significantly decreased in the *C*max concentrations of fluconazole (FLUCO) and voriconazole (VORI) compared with the negative control in medium and plasma (Figure 1(a)). A further significant decrease in higher concentrations was only seen after incubation with anidulafungin (ANI) down to zero cells in the 10x *C*max concentration (data not shown).

The vitality was decreased in the *C*max concentration after incubation with all antimycotics in medium and plasma (Figure 1(b)). Higher concentrations of ANI led to a markedly dose-dependent decrease of vitality (Figure 1(c)), which was not seen after incubations with the other antimycotics.

### 3.2. Anidulafungin and Fluconazole Cause Loss of Cell Integrity (LDH)

**Figure 2:**
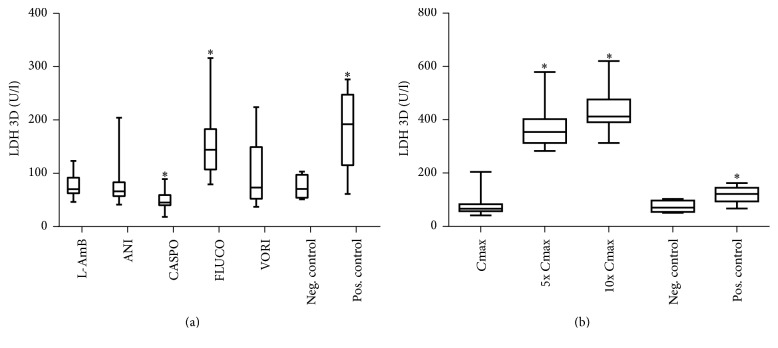
Effects of antimycotics agents on lactate dehydrogenase (LDH) release [U/l] in medium. Lactate dehydrogenase concentration in cultured C3A cells was measured after 72 hours of treatment with 5.7 mM liposomal amphotericerin B, 2.25 mM anidulafungin, 0.2 mM, caspofungin, 4.5 mM fluconazole, 0.25 mM voriconazole and untreated (negative control) and 15.25 mM APAP (positive control) (a). LDH was further measured in different doses of anidulafungin (b). Results are expressed as median and 25th/75th percentile (*n* = 20 biological and *n* = 40 technical repeats). *∗* indicates significance of *p* < 0.05 against the negative control.

Significant increases of lactate dehydrogenase (LDH) in the *C*max concentrations were only seen after incubation with FLUCO compared with the negative control in medium and plasma (Figure 2(a)). After incubation with caspofungin (CASPO) significant lower values of LDH were measured. Dose-dependent effects were observed after incubation with ANI; Figure 2(b) shows the significant increase of LDH after incubation with ANI in higher concentrations (Figure 2(b)). At 3 days, the differences of LDH between the groups were greater than those at 6 days, but the same trends were seen (data not shown).

### 3.3. Effects of Antimycotic Drugs on the Activity of Mitochondrial Dehydrogenases in Hepatocytes

**Figure 3:**
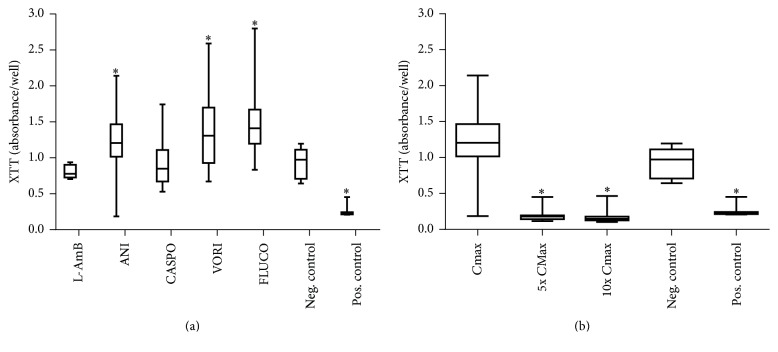
Effects of antimycotics agents on the activity of mitochondrial dehydrogenases: XTT test [absorbance/well] in medium. XTT assay of treated hepatocytes with 5.7 mM liposomal amphotericerin B, 2.25 mM anidulafungin, 0.2 mM caspofungin, 4.5 mM fluconazole, and 0.25 mM voriconazole and untreated (negative control) and 15.25 mM APAP (positive control). LDH concentrations in medium of antifungal treated hepatocytes after 144 hours (a) and dose-dependent effects of anidulafungin incubated hepatocytes (b). Results are expressed as median and 25th/75th percentile (*n* = 20 biological and *n* = 40 technical repeats). *∗* indicates significance of *p* < 0.05 against the negative control.

The activity of mitochondrial dehydrogenases was examined by the XTT test. Displayed in Figure 3(a), a significant increase was seen after incubation with ANI, FLUCO, and VORI in the *C*max concentrations in medium and plasma compared with the negative control. In contrast, incubation with ANI in higher concentrations (5x *C*max, 10x *C*max) led to significantly lower values in the XXT test (Figure 3(b)).

### 3.4. Liposomal Amphotericerin B Induced an Increase of CYP1A2 Activity

**Figure 4:**
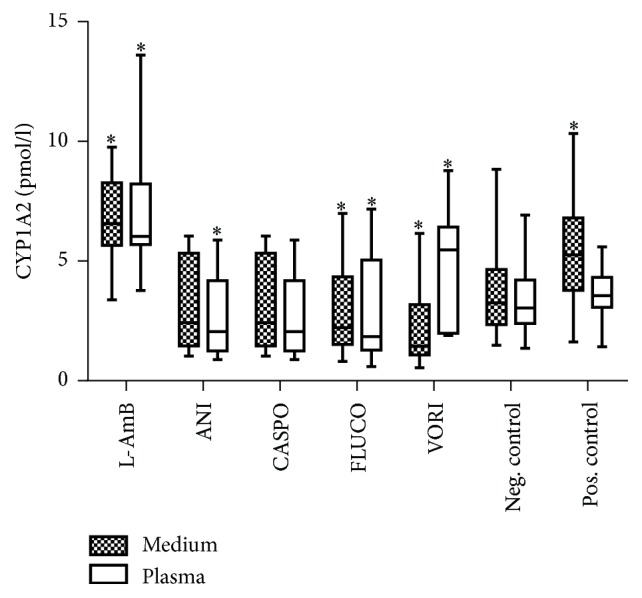
Effects of antimycotics agents on the cytochrome 1A2 activity (7-ethoxyresorufin O-deethylation and conversion of ethoxyresorufin to resorufin) [pmol/l] in medium and plasma. Cells in culture were treated with 5.7 mM liposomal amphotericerin B, 2.25 mM anidulafungin, 0.2 mM caspofungin, 4.5 mM fluconazole, and 0.25 mM voriconazole, and untreated (negative control) and 15.25 mM APAP (positive control). Results are expressed as median and 25th/75th percentile (*n* = 20 biological and *n* = 40 technical repeats). *∗* indicates significance of *p* < 0.05 against the negative control.

Liposomal amphotericerin B (L-AmB) and the positive control led to an increase of the cytochrome 1A2 activity; ANI, FLUCO, and VORI led to a significant decrease in the *C*max concentrations compared with the negative control in medium. Interestingly, incubation of the *C*max concentration of VORI with the test cells in plasma increased the cytochrome 1A2 activity (Figure 4). Dose-dependent effects were not seen.

### 3.5. Effects of Antimycotics on Synthesis of Albumin

**Figure 5:**
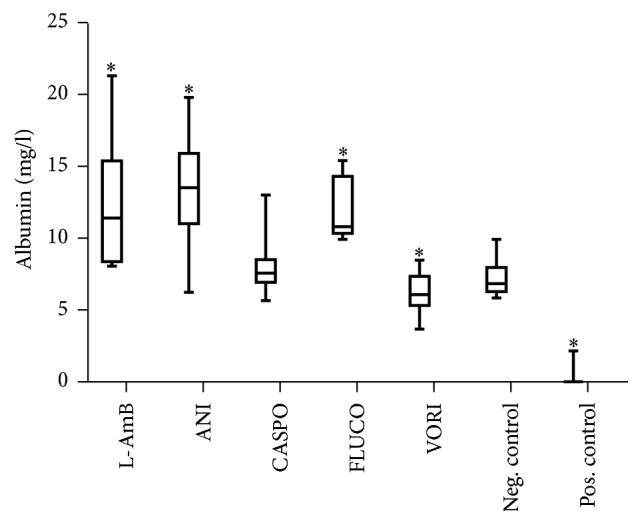
Release of albumin [mg/l] in medium. Treatments of cells with *C*max concentrations of 5.7 mM liposomal amphotericerin B, 2.25 mM anidulafungin, 0.2 mM caspofungin, 4.5 mM fluconazole, 0.25 mM voriconazole, and untreated (negative control) and 15.25 mM APAP (positive control). Results are expressed as median and 25th/75th percentile, *n* = 20 (biological and technical repeats). *∗* indicates *p* < 0.05 against the negative control.

Significant increased values of albumin were detected in the cell culture supernatant after incubation with the *C*max concentrations of ANI, L-AmB, and FLUCO; in contrast, incubation with VORI in the *C*max concentration led to a significant decrease of the synthesis of albumin in medium and plasma (Figure 5). A dose-dependent decrease of albumin was observed after incubation with FLUCO and ANI; especially at higher concentrations of ANI (5x *C*max and 10x *C*max) synthesis of albumin decreased by 100% versus controls (data not shown).

### 3.6. Higher Concentrations of Anidulafungin Induced Cell Death (TUNEL Assay)

The part of apoptotic or necrotic cells (DNA fragmentation) was low (5%) after incubation with the *C*max concentrations of all used antimycotics and was comparable with the values of the negative control in medium and plasma (data not shown). A concentration-dependent influence on DNA fragmentation was only seen after incubation with ANI: at the 5x *C*max concentration in 32.8% of the hepatocytes apoptotic or necrotic cells determined and at the 10x *C*max concentration in 58.5% determined (*C*max concentration-ANI: 2.6% apoptotic cells).

## 4. Discussions

In our study we investigated the in vitro hepatotoxicity of antimycotics in clinical relevant concentrations (*C*max; 5x *C*max; 10x *C*max) [23–27] used for systemic infections with an established in vitro cytotoxicity screening model based on HepG2/C3A cells [19–22]. All drugs were tested in cell culture medium (with 10% FBS) and plasma from healthy volunteers (pooled plasma) as a control. The idea behind this was to evaluate the influence of the different plasma protein bindings on the cytotoxicity of the used drugs. Only the CYP 1A2 activity in voriconazole-incubated cells was markedly higher in the control than in the medium. In all other results we did not see any difference between both approaches. We tested the viability of cells (XTT test, LDH release, and trypan blue staining), the synthesis of microalbumin, the cytochrome 1A2 activity, and the cell death (TUNEL assay). The use of XTT is an established test for the activity of mitochondrial dehydrogenases and the EROD-test for the activity of cytochrome 1A2, mainly involved in the metabolism of exogenous substances. In both tests, an increase as well as a decrease of activity is a marker for toxic reactions of cells on different agents [30–33].

The human hepatocellular carcinoma cell line HepG2/C3A, a subclone of the hepatoma-derived HepG2 cell line, is an effective in vitro liver model that has been used to identify and evaluate compounds that may have hepatotoxic properties. The biosynthetic capabilities of C3A, similar to primary human hepatocytes, such as the production of many liver-specific plasma proteins [28], the presence of a functional cytochrome P450 toxin-processing enzyme system after stimulation [34, 35], and glucuronic- and sulfate-conjugation abilities were investigated and confirmed by independent investigators. Therefore, the cell line has the ability to carry out normal biotransformation reactions essential for the detoxification process. Flynn and Ferguson [36] and Liu et al. [37] treated HepG2/C3A cells with single, purified compounds and used a large number of biologically relevant assays to estimate the hepatotoxic potential of chemicals.

The C3A cell line does not completely mimic the behavior of liver lobule; nevertheless, C3A cells are a useful model for the predictive modeling of hepatotoxic agents [34], microfluidic devices [35], metabolome and tissue engineering approaches including 3D spheroid formation [38], and cell therapeutic applications, such as the extracorporeal liver assist device (ELAD) system [39].

The testing of anidulafungin, caspofungin, and fluconazole with C3A cells compared with primary isolated human hepatocytes provided similar results with the same trends focusing on cytochrome 1A2 activity, vitality, and activity of mitochondrial dehydrogenase (XTT or MTT test; own data, not shown).

Antifungal agents have been implicated in numerous cases of hepatotoxicity. Of all reported drug-related hepatotoxicity events, nearly 3% are mediated by antifungal agents [12]. On the other hand, the incidence of invasive fungal infections has increased steadily over the two decades [40]. The infections are characterized by high mortality and morbidity in immunocompromised patients. The most common species of human fungal pathogens are* Aspergillus fumigatus* (50–90%) and* Candida albicans* (20–40%) [41]. Current treatment strategies for these infections have limitations in their spectrum of activity, drug interactions, pharmacokinetics, and pharmacodynamics properties, toxicity (dosing safety profile), and expense [42].

The risk of developing hepatotoxicity by antifungal agents is multifactorial; factors are preexisting liver disease, drug-drug interactions, the chemical properties of the antifungal agent, comorbidities, environmental factors (e.g., alcohol or the use of concomitant hepatotoxic drugs), genetic factors, the severity and treatment of the underlying disease, and liver involvement by the fungal infection [12, 43].

Liver injury caused by antifungal agents may vary from slight and asymptomatic abnormalities in liver enzymes to hepatotoxicity that occurs as potentially fatal fulminant liver failure [44]. Clinically significant liver injuries manifest mostly as acute hepatocellular, cholestatic, or mixed hepatocellular and cholestatic reactions [45]. The exact mechanisms of the toxicity of many of the antimycotics are unknown [46]; however, there has been no systematic evaluation of the liver toxicity associated with these treatments.

Liposomal amphotericin B (L-AmB) showed in our study with the C3A cell line a mild cytotoxicity on hepatocytes in all concentrations. A concentration-dependent manifestation of toxicity was not seen. The general toxicity of amphotericin B is known; however, the lipid formulation demonstrated an improved safety profile compared to conventional amphotericin B. It is still unclear how frequently main side effects occur. Severe cases of L-AmB induced hepatotoxicity have been reported, but overall clinically apparent discontinuation of therapy and hepatotoxicity are rare [47]. Fischer et al. reported that 32% of hematopoietic stem cell transplantation (HSCT) patients, who received cumulative doses > 3.7 g, developed a dose-dependent hepatotoxicity [47]. Wang et al. showed in a meta-analysis that the risk was only 0.7% for all lipid forms of amphotericerin B for a treatment discontinuation due to liver injury [44]. The L-AmB-induced hepatotoxicity in animal experiments is probably caused by the lipid component of the drug, but this has not yet been confirmed in humans (data in humans are lacking) [48].

The azoles fluconazole and voriconazole showed a higher toxic potential on hepatocytes in all concentrations in our study; especially in voriconazole-incubated cells these effects were more pronounced. Interestingly, these effects were not dose-dependent.

The incidence of mild transient liver damage associated with azole drugs is 2–12% [47]. A clear dose or time course relationship is not known for the azoles, but the use of voriconazole leads to liver damage. The toxicity is usually hepatocellular but can rarely occur as cholestatic or both [49, 50]. Predominantly, the cholestatic or the mixed pattern of liver damage recurs with the restoration of fluconazole treatment [44, 51]. All azole antifungals are metabolized in the liver; while fluconazole is metabolized only minimally, itraconazole, voriconazole, and posaconazole are highly dependent on the metabolism for drug elimination [15]. All cases of hepatocellular-cholestatic liver injury were observed by fluconazole-treatments [52]. The mechanism of liver damage is unknown, but it is different from other triazole antifungal drugs, because fluconazole is excreted primarily in the urine and is the least potent as substrate or inhibitor of hepatic metabolism. Voriconazole-induced severe hepatotoxicity and treatment have been reported; however, hepatotoxicity was reversible, when ending therapy. Denning et al. reported in 15% of adult patients, receiving voriconazole, about abnormalities in liver enzymes with treatment discontinuation [53]. In patients, the observed pattern of liver damage was not uniform; it included a mixed hepatocellular-cholestatic, hepatocellular, or cholestatic injury [53]. For all patients a careful monitoring of liver enzymes is recommended receiving azole therapy. Abnormalities in liver enzymes, although reversible and rare after termination of treatment, are the most common laboratory abnormalities in voriconazole-treated patients [54]. Voriconazole appears to have a higher risk of liver damage than other modern antifungals [55, 56]. In summary, voriconazole should be used with caution in patients with liver dysfunction or liver failure [54].

In this study, we observed a mild cytotoxicity on hepatocytes of echinocandin (anidulafungin, caspofungin) in the *C*_max_ concentration. In contrast, higher concentrations (5x *C*max, 10x *C*max) of anidulafungin led to severe impairment of vitality, viability, and function of hepatocytes. In our study the lowest level of cytotoxicity on hepatocytes was found after incubation with caspofungin. Echinocandins have a low risk of hepatotoxicity and increased liver values; however, there are warnings in the prescribing information for all drugs of the group that hepatotoxicity may occur [57]. Clinically obvious hepatotoxicity appears only in isolated cases. Patients treated with the echinocandins observed transient increases in the liver enzymes that typically return to the baseline after the withdrawal of the therapy. Caspofungin can cause a higher rate of serum liver injury than micafungin or anidulafungin [58]. The cause of liver enzyme elevations during echinocandin therapy is partly unknown [44]. Echinocandins have been used safely in patients with preexisting hepatic impairment, namely, caspofungin in patients with chronic liver disease and postliver transplantation. Micafungin and anidulafungin have been used in patients with hepatic impairment; no worsening of the disease was observed [59, 60].

At the moment, it is still unclear if the hepatotoxic effect of the echinocandins may aggravate acute liver damage. Anidulafungin is not hepatically metabolized in contrast to caspofungin and micafungin [61]. All of them undergo a nonenzymatic chemical degradation to form a ring-opened product under neutral to basic conditions. The reactive intermediate formation is evident for the hepatotoxic potential; particularly anidulafungin generates a reactive intermediate able to trap glutathione [62–64].

Liver enzyme anomalies under caspofungin therapy are rare [65, 66]. The coadministration of cyclosporine increased caspofungin exposure and has been associated with elevated liver transaminase levels in volunteers for a short time [67]. Compared to treatments with fluconazole and AmB, the incidence of liver damage in caspofungin-treated patients is lower. In a controlled study comparing caspofungin and AmB in 224 adults with invasive candidiasis, patients with caspofungin-treatment showed a lower ALT (3.7% versus 8.1%), ALP (8.3% versus 15.6%), and bilirubin (2.8% versus 8.9%) serum levels abnormalities and no severe hepatic side effects [68]. In a study of 83 patients with invasive aspergillosis treated with caspofungin for over 20 days, ALT elevations occurred only in one patient and no serious drug-related hepatic side effects were reported [69]. Higher drug levels of caspofungin in patients with moderate liver insufficiency were found in nearly 75% of cases. A dose reduction to 35 mg daily, after the 70 mg administration dose, is recommended in the package insert; however, clinical experience with the use of caspofungin in patients with severe hepatic impairment (Child-Pugh score of > 9) is very low [65].

The use of anidulafungin is rarely associated with adverse events and does not appear to be significantly hepatotoxic [57, 70]. Saliba et al. found in a randomized, double blind study of anidulafungin versus fluconazole for the treatment of esophageal candidiasis an increase of liver enzymes including gamma glutamyl transpeptidase (*γ*-GT) (1.3% in both groups) and AST (0.3% anidulafungin versus 2.3% of patients treated with fluconazole) [70]. In contrast to our results, according to the package insert no dosing adjustments of anidulafungin are necessary in patients with mild, moderate, or severe hepatic impairment [71].

## 5. Conclusion

In our in vitro liver model we assessed cytotoxicity of antimycotics. Concerning cytotoxic safety assessment, liposomal amphotericin B and caspofungin showed mild hepatotoxicity. The azole fluconazole and voriconazole showed a higher hepatotoxic potential; anidulafungin showed in higher concentrations (5x *C*max, 10x *C*max) a severe impairment of hepatocyte vitality, viability, and function. The mechanisms for the hepatotoxicity are unclear and need further investigations in suitable in vitro and in vivo models.

Azoles used for the therapy of systemic infections should be given with caution in patients with liver insufficiency and liver failure or high risk for this. Therefore, therapeutic drug monitoring (TDM), particularly during therapy with anidulafungin, may help to optimize the safety of therapy.
